# Correlation analysis between lower limb muscle architectures and cycling power via ultrasonography

**DOI:** 10.1038/s41598-021-84870-x

**Published:** 2021-03-08

**Authors:** Hyung-Jin Lee, Kang-Woo Lee, Kurokawa Takeshi, Yong-Woo Lee, Hee-Jin Kim

**Affiliations:** 1 Division in Anatomy and Developmental Biology, Department of Oral Biology, Human Identification Research Institute, BK21 PLUS Project, Yonsei University College of Dentistry, Room 6 01, 50 Yonsei-ro, Seodaemun-gu, Seoul, 03722 Republic of Korea; 2 National Institute of Fitness & Sports in KANOYA (Coaching of Sports and Budo), 1, Shiromizucho, Kanoya City, Kagoshima Pre. 891-2311 Japan; 3 Department of Physical Education, College of Sports Science, Korea National Sport University, Yangjaedaero 1239, Songpa-gu, Seoul, 05541 Republic of Korea

**Keywords:** Musculoskeletal system, Ultrasonography

## Abstract

The primary purpose was to examine the relationship between the muscle architectural characteristics of short and long-distance cyclist—including muscle thickness, fascicle angle, and fascicle length—of the anterior thigh and posterior leg and its impact in 20-s cycling power. The secondary purpose was to clarify the muscle variables that predict the cycling power by using ultrasonography to measure the muscle architectural characteristics. Twenty-four varsity cyclists participated in this study, of whom 12 were short-distance cyclists and 12 were long-distance cyclists. B-mode ultrasonography was used to measure muscle architecture parameters. A cycle ergometer was used to measure the cycling power. The rectus femoris, vastus medialis, and medial head of gastrocnemius were significantly thicker in short-distance cyclists than in long-distance cyclists at every site (*p* < 0.05). Our analysis revealed that the rectus femoris fascicle length at the 30% level of the thigh was a significant independent predictor of the 20-s cycling power in short-distance cyclists, while the rectus femoris fascicle angle at the 50% level was that of the 20-s cycling power in long-distance cyclists. These findings highlight the significance of rectus femoris muscle architecture to cycling power.

## Introduction

The underlying anatomical and physiological determinants of the performance of a cyclist include pedaling rate, muscle size and architecture, knee extension strength, distribution of muscle fiber types, cycling position, fatigue, and cardiovascular endurance^1–8^.

Muscle architectural characteristics including muscle thickness, muscle volume, fascicle angle, and fascicle length are strongly correlated with the maximum muscle strength and power^9–14^. The maximum voluntary isometric force is proportional to the physiological cross-sectional area, which can be regarded as the sum of the cross-sectional areas of all muscle fibers^15^. The fascicle angle refers to the directional angle between the muscle fibers and the tendon, and is 0° when the muscle fibers attach to the tendon in parallel. Such an arrangement will ensure that the muscle will transmit 100% of the force generated during contraction to the tendon, whereas a pennate muscle group with a 30° fascicle angle delivers only about 86% of the contractile force (The cosine 30° is 0.86)^15,16^. In general, pennate muscles have a larger physiological cross-sectional area and an ability to generate large forces since there are more fibers packed into a given muscle length, giving rise to generation of large forces and rotation of the muscle fibers during contraction^15^.

The fascicle length affects the muscle shortening velocity, with a longer muscle fascicle resulting in faster muscle shortening^10,17,18^. The impact of the number of sarcomeres in parallel and in series on cross-sectional area and muscle fiber length can affect the cell’s force-generating ability^15^. Although muscle architecture is one of the most-reliable and important key parameters in predicting human movement and sports performance^1,5,6,8,9,12–14,16–23^ (e.g. muscle force generation), previous studies of cyclists have mostly focused on the pedaling technique, the muscular activity during ergometer and uphill cycling in electromyography (EMG), and the knee extension torque^2,8,21,24^. Few studies have employed anatomical and physiological approaches with ultrasonography (US) to determine how the muscles of the anterior thigh and the posterior leg contribute to cycling power.

Architectural characteristics of muscles such as their thickness, fascicle angle, and fascicle length may vary with the exercise type and duration^25,26^. There are several reports stating that the muscle thickness, fascicle angle, and fascicle length tend to change significantly depending on the training period or a specific sporting event, resulting in specialized muscle function for the corresponding exercise^14,17–20,27,28^. For example, compared to nonathletes, experienced rowing athletes and cyclists exhibit larger anatomical cross-sectional area of the quadriceps femoris (QF), which is induced by their repetitive multijoint leg extensions and pedaling technique^13,29^. Since the muscle development profiles differ between athletes and nonathletes, significantly different muscle morphologies and functions are also found within the QF and another muscle groups^12,13,18,20,29,30^. Therefore, it is essential to determine which muscles and their architectural characteristics are strongly correlated with—or are strong contributors to—cycling performance or cycling power. A better understanding of these factors would lead to effective exercise prescriptions to improve cycling power and, consequently, to maximize cycling performance.

The gold standard for analyzing the architectural characteristics of human muscle is magnetic resonance imaging (MRI), but US has been actively used since 1990 to examine muscle architectural characteristics. Various studies have analyzed how the architectures of the QF, gastrocnemius, pectoralis major, and latissimus dorsi of swimmers, soccer players, sprinters, and distance runners are related to their sport performance or performance records^14,17,18,23^. However, none of these studies have attempted to reveal which muscle is the strongest predictor both anatomically and physiologically of the cycling power. Therefore, the primary purpose of the present study was to determine the muscle architectural characteristics of short- and long-distance cyclists and examine the relationship between these characteristics—including muscle thickness, fascicle angle, and fascicle length—of the anterior thigh and posterior leg of cyclists and the 20-s cycling power. The secondary purpose was to clarify the muscle variables that predict the cycling power by using US to measure the muscle architectural characteristics.

## Results

The values of body height, body mass, and cycling power of short- and long-distance cyclists are summarized in Table 1.

**Table 1:** Summary of measured values of the participants.

Variables	S (*N* = 12)	L (*N* = 12)	Range
Height (cm)	174.5 ± 4.4	174.5 ± 4.6	[165–183]
Body mass (kg)	76.5 ± 7.5*	67.8 ± 6.6	[56–90]
20 s cycling power (W)	1077.0 ± 102.0*	907.6 ± 99.7	[769.6–1217.5]

S, short-distance cyclist; L, long-distance cyclists; *significant differences between short- and long-distance cyclists, *p < 0.05.

### Difference of muscle architecture between short- and long-distance cyclists

The rectus femoris (RF), vastus medialis (VM), and medial head of the gastrocnemius (Gm) were significantly thicker in short-distance cyclists than in long-distance cyclists at every site (*p* < 0.05). The fascicle lengths of the vastus lateralis (VL) at the 50% level of the anterior thigh region and of the Gm at the 30% level of the posterior leg region were significantly longer in short-distance cyclists than in long-distance cyclists (*p* < 0.05). The fascicle angle of the RF was significantly larger in short-distance cyclists than in long-distance cyclists measured at the 50% level of the anterior thigh region (*p* < 0.05) (Table 2).

**Table 2:** Muscle architectural characteristics of cyclists.

Variables	Group	Measured location and correspond values				
	All (*N* = 24) S (*N* = 12) L (*N* = 12)	Mean ± SD				
**Muscle thickness (mm)**		20%	30%	50%	70%	90%
RF^**†**^	All		26.0 ± 4.0^**a**^	26.4 ± 4.6^**a**^	19.9 ± 5.8^**b**^	
	S		27.9 ± 3.3^§^	28.8 ± 5.0^§^	23.0 ± 5.4^§^	
	L		23.9 ± 3.6	22.9 ± 2.5	16.9 ± 4.7	
VI	All		22.3 ± 4.3	20.6 ± 4.6	17.2 ± 3.6^a,b^	
	S		23.3 ± 4.6	22.7 ± 3.9	17.4 ± 2.9	
	L		21.2 ± 3.9	19.2 ± 4.9	17.0 ± 4.4	
VL^**†**^	All		28.5 ± 4.8^**a**^	28.0 ± 5.3^**a**^	22.4 ± 4.7^**b**^	
	S		28.9 ± 5.2	29.2 ± 6.4	24.0 ± 5.2	
	L		28.0 ± 4.5	26.9 ± 3.9	20.7 ± 3.7	
VM^‡^	All				34.7 ± 8.1^**A**^	29.8 ± 5.9^B^
	S				39.3 ± 6.6^§^	32.7 ± 5.3^§^
	L				30.1 ± 6.8	26.6 ± 4.8
Gm^‡^	All	20.0 ± 3.1	18.2 ± 4.5			
	S	21.5 ± 3.5^§^	20.9 ± 4.1^§^			
	L	18.5 ± 1.9	15.9 ± 3.2			
Gl^‡^	All	15.3 ± 3.2	13.3 ± 4.0			
	S	15.4 ± 2.9	13.0 ± 2.6			
	L	15.2 ± 3.7	13.7 ± 5.2			
**Fascicle angle **(°)						
RF^†^	All		13.2 ± 3.9^**a**^	10.3 ± 1.7^**a**^	9.5 ± 4.2^**b**^	
	S		13.2 ± 4.2	9.9 ± 1.2	10.1 ± 5.9	
	L		13.3 ± 3.8	10.8 ± 2.2	8.9 ± 5.9	
VI	All		10.2 ± 2.4^**a**^	8.1 ± 2.4^**a**^	6.8 ± 1.9^**b**^	
	S		10.0 ± 2.4	9.1 ± 2.6	6.7 ± 1.8	
	L		10.4 ± 2.5	6.8 ± 1.7	6.8 ± 2.2	
VL^†^	All		13.4 ± 2.5	13.5 ± 3.4	15.2 ± 3.4	
	S		13.0 ± 1.9	14.5 ± .4.7^§^	15.3 ± 4.3	
	L		13.6 ± 3.0	12.7 ± 2.0	15.1 ± 2.6	
Gm^‡^	All	18.1 ± 3.0	18.8 ± 3.6			
	S	18.7 ± 3.1	20.3 ± 3.7^§^			
	L	17.5 ± 2.9	17.2 ± 2.8			
Gl^†^	All	13.4 ± 2.5	13.9 ± 2.4			
	S	13.9 ± 3.1	13.9 ± 2.3			
	L	12.9 ± 1.8	13.9 ± 2.6			
**Fascicle length (mm)**						
RF^†^	All		121.5 ± 40.5^a^	153.1 ± 32.0^b^	137.5 ± 43.4^a^	
	S		133.3 ± 47.9	167.7 ± 21.1^§^	147.9 ± 32.6	
	L		110.8 ± 31.1	134.5 ± 35.1	128.7 ± 51.8	
VI	All		128.7 ± 37.9	158.5 ± 62.4	153.6 ± 44.5	
	S		129.7 ± 44.5	156.7 ± 75.2	158.1 ± 48.7	
	L		127.6 ± 30.5	160.6 ± 46.5	149.0 ± 41.8	
VL^†^	All		128.5 ± 30.1^**a**^	131.4 ± 40.9^**a**^	89.1 ± 20.8^**b**^	
	S		138.1 ± 38.2	133.7 ± 49.0	97.0 ± 20.6	
	L		120.8 ± 20.2^**a**^	129.6 ± 36.6^**a**^	82.0 ± 19.0^**b**^	
Gm^†^	All	65.9 ± 10.9^**A**^	56.7 ± 9.7^**B**^			
	S	70.4 ± 10.3	57.2 ± 11.5			
	L	61.2 ± 8.9	56.2 ± 7.7			
Gl^†^	All	66.5 ± 14.7^**A**^	54.6 ± 15.3^**B**^			
	S	66.9 ± 15.9	54.5 ± 19.1			
	L	66.0 ± 14.2	54.7 ± 11.8			

RF, rectus femoris; VI, vastus intermedius; VM, vastus medialis; VL, vastus lateralis; Gm, medial head of gastrocnemius; Gl, lateral head of gastrocnemius; S, short-distance cyclist; L, long-distance cyclist. ^†^One-way ANOVA, mean (95% confidence interval), within the same row, the different small letters indicate significant differences between the measured sites for each muscle in short- and long-distance cyclists by Tukey’s post-hoc test. ^‡^Paired t-test with Bonferroni correction, within same row, the different capital letters indicate significant differences between each measured site for VM, Gm, and Gl and short- and long-distance cyclists. ^§^Independent t-test, within the same column, the section signs indicate significant differences between short- and long-distance cyclists. The percentages (30, 50, 70, and 90 of the RF, VI, VL, and VM) represent the scanning sites between the greater trochanter and the lateral epicondyle of the femur. The percentages (20 and 30 of Gm and Gl) represent the scanning sites between the fibular head and the lateral malleolus.

### Correlations between muscle architectural characteristics and 20-s mean anaerobic power

The body mass, fascicle angle of the RF at the 50% level, fascicle length of the Gm at the 20% level, and fascicle length of the Gm at the 30% level were strongly correlated with the 20-s cycling power in long-distance cyclists (*r* = 0.73, 0.93, 0.63, and 0.70, respectively). The muscle thicknesses of the RF at the 30% and 50% levels, of the VL at the 50% and 70% levels, and of the Gm at the 30% level, as well as the fascicle lengths of the RF at the 30% level, of the Gm at the 20% and 30% levels, and of the lateral head of the gastrocnemius (Gl) at the 30% level were strongly correlated with the 20-s cycling power in short-distance cyclists (Table 3).

**Table 3:** Coefficients for Pearson’s correlations between cycling power and muscle architectural characteristics (i.e., muscle thickness, fascicle angle, and fascicle length).

Variables	20 s mean power Group		
	All (*N* = 24)	S (*N* = 12)	L (*N* = 12)
**Muscle thickness (mm)**			
RF 30%	.45*	.64*	.05
RF 50%	.58**	.62*	-.18
VL 50%	.48*	.66*	.23
VL 70%	.55**	.60*	.24
Gm 20%	.61**	.35	.07
Gm 30%	.50*	.59*	-.08
**Fascicle angle (°)**			
RF 50%	.11	-.08	.93**
**Fascicle length (mm)**			
RF 30%	.52*	.81*	.26
Gm 20%	.62**	.72*	.63*
Gm 30%	.59**	.79**	.70*
Gl 30%	.13	.74*	-.18

The percentages (30, 50, 70, and 90 of RF, VL, VI, and VM) represent the scanning sites between the greater trochanter and the lateral epicondyle of the femur. The percentages (20 and 30 of Gm and Gl) represent the scanning sites between the fibular head and the lateral malleolus. Significantly different, **p < 0.01, *p < 0.05.

### Prediction of 20-s mean anaerobic power according to muscle architectural characteristics

**Figure 1:**
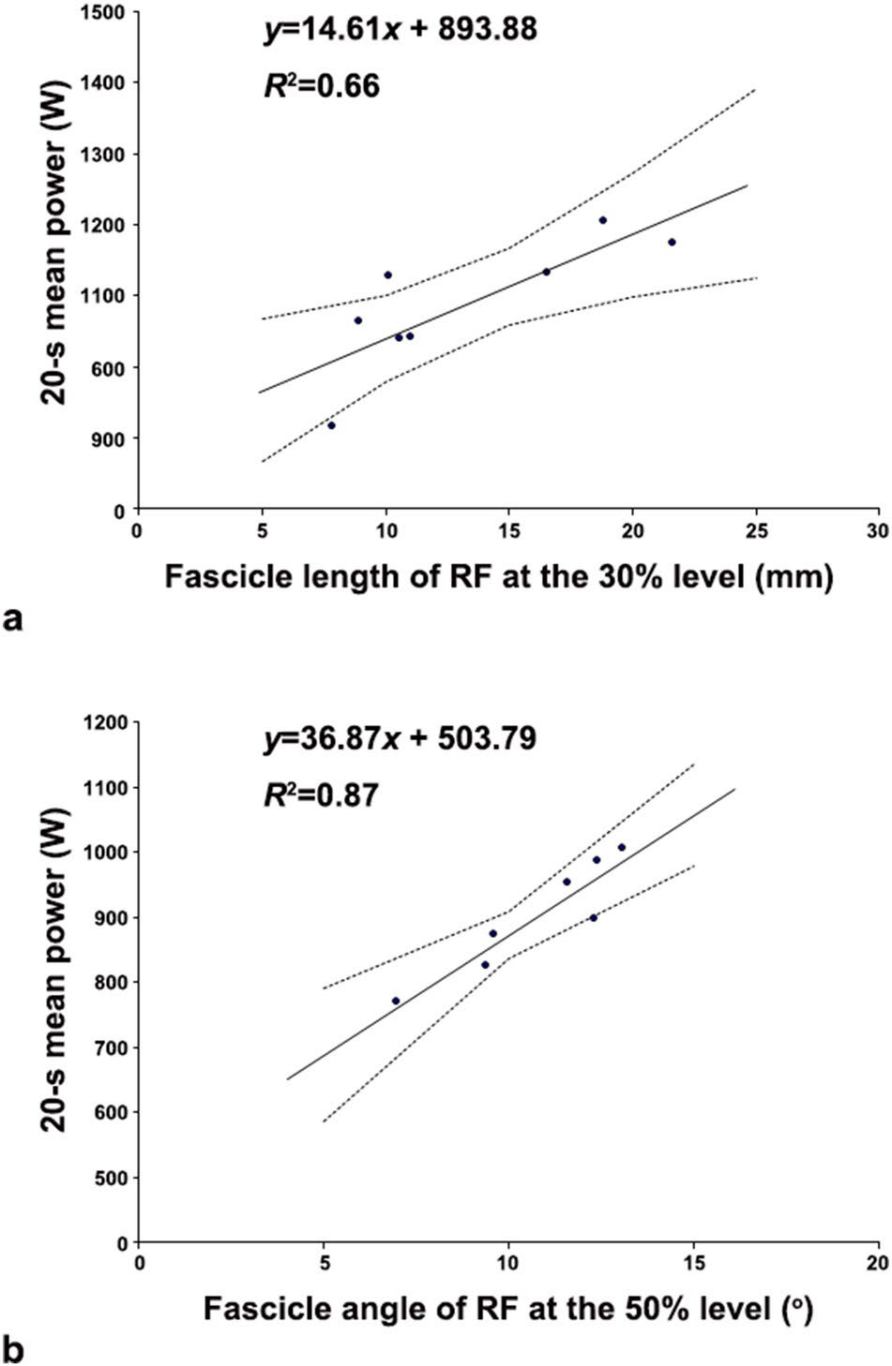
The fascicle length of the RF at the 30% level of the thigh region explained a large proportion of the variance in the 20-s cycling power in short-distance cyclists (**a**). The fascicle angle of the RF at the 50% level of the thigh region explained a large proportion of the variance in the 20-s cycling power in long-distance cyclists (**b**). Data points in A and B are from eight short-distance cyclists and seven long-distance cyclists, respectively. The two dotted lines in each graph indicate the 95% confidence interval, and the solid line indicates the best linear fit to the data points.

Stepwise multiple regression analysis revealed that the RF fascicle length at the 30% level was a significant independent predictor of the 20-s cycling power (*R*^2^ = 0.66, *n* = 8) in short-distance cyclists, explaining 66% of the variance (Fig. 1A), while the RF fascicle angle at the 50% level was a significant independent predictor of the 20-s cycling power (*R*^2^ = 0.87, *n* = 7) in long-distance cyclists, explaining 87% of the variance (Fig. 1B).

## Discussion

This study investigated the muscle architectural characteristics of short- and long-distance cyclists and examined the relationships of the muscle architectural characteristics of the QF and the gastrocnemius with cycling power. The stepwise multiple regression analysis showed that the fascicle length and fascicle angle of the RF explained a large proportion of the variance in the 20-s cycling power in short-distance and long-distance cyclists, respectively. Our observations uniquely demonstrate both the importance of the detailed QF architectural characteristics of cyclists and the different contributions of muscle architectural characteristics to cycling power for both short- and long-distance cyclists.

The following muscles were significantly thicker in short-distance cyclists than in long-distance cyclists: RF (16.7%, 26.3%, and 36% thicker at proximal, mid-thigh, and distal sites, respectively), VM (30.6% and 22.9% thicker at proximal and distal sites, respectively), and Gm (16.2% and 31.5% thicker at proximal and distal sites, respectively). Also, the fascicle angles of the VL (14.2% larger at the mid-thigh site) and Gm (18% larger at the distal site) were larger and the fascicle length of the RF (24.7% longer at the mid-thigh site) was longer in short-distance cyclists than in long-distance cyclists. Kumagai et al. reported that nonuniform quadriceps femoris and hamstring muscle shapes (from black and white college football players) were correlated with faster 40-yards sprint times^23^. In another study, Abe et al. reported that male sprinters had significantly greater muscle thickness in the proximal and mid portions but not in the distal portion of the anterior thigh (i.e., quadriceps femoris muscle) than distance runners^17^. Based on these studies, they hypothesized that muscle shape could be associated with better sprint performance. These implications were supported by the study of Narici et al. in which changes in the cross-sectional area along the length of the quadriceps femoris muscles were observed after high-intensity weight training^31^. They suggested that varying degrees of hypertrophic response could exist in each of the constituent muscles of the quadriceps. Even though there is no specific analysis or study concerning the functional ability of nonuniform architectural differences, we could hypothesize that different muscle shapes might correlate with cycling performance; however, more specific and meticulous work is needed to elucidate this phenomenon.

The main cycling events for short-distance cyclists are the 200-m sprint, keirin, and 1000-m time trial on the track. For the 200-m sprint, the peak power was found to be about 1020 W, with peak and average pedaling cadences of 150 revolutions per minute (rpm) and 142 rpm, respectively. For the 1000-m time trial, the peak power was 1799 W and the average cadence was 127 rpm^32^. As mentioned above, most sprinters produce large forces for less than 10 s, and many previous studies have revealed that aspects of the muscle architecture such as muscle thickness, fascicle angle, and fascicle length are reliable parameters for evaluating muscle function and strength. Abe et al. reported that the muscle thickness was significantly related to muscle strength and that elite sprinters have significantly thicker muscles in the upper portion of the anterior thigh compared to distance runners (by 13.4% and 10.7% at 30% and 50% along the thigh, respectively). Also, the muscle thickness and fascicle length of the gastrocnemius were significantly larger in sprinters than in distance runners^17^. These different architectural characteristics of the muscle are associated with faster sprint performances. Lee et al. also demonstrated that the muscles in the anterior thigh and abdominal regions were thicker in short-distance cyclists than in long-distance cyclists^1^. These differences in muscle architecture could be attributed to short-distance cyclists generating larger forces over shorter times and shorter distances compared to long-distance cyclists, and resulted in the 20-s cycling power being higher in short-distance cyclists in the present study.

The findings of numerous studies of athletes and untrained control subjects that have compared muscle architectures suggest that competitive sports activities significantly affect characteristics of the muscle architecture^12,14,18,20,23,29^. Furthermore, some reports suggested that sports performance is correlated with the fascicle arrangement within a muscle, while longitudinal studies have produced direct evidence that the arrangements of the fascicle adapt to various types of training^20,27^. Based on the results of the present and previous studies, it can be suggested that coaches and trainers should establish different types of resistance and cycle training that focus on strengthening specific muscles in short- and long-distance cyclists wanting to improve their cycling power.

The muscles of the leg are extremely important for power output and the propulsion phase of cycling. Strong knee flexion and hip flexion occur during the upstroke pedaling movement, while strong knee extension occurs during the downstroke pedaling movement^2,4,22,33^. The muscle that is most strongly correlated with knee joint extension is obviously the QF, and this muscle is highly developed and hypertrophied in cyclists. Considering that the VL and VM contribute greatly to cycling, numerous studies have attempted to reveal their activation and recruitment patterns and to determine which muscle most affects the cycling power^4,21,22,33^. Lee and colleagues recently used US to examine the correlations between the thickness of the QF at a single site and the 5-s, 30-s, and 3-min cycling powers. Those authors found that the VL and VM muscle thicknesses were significantly correlated with the 5-s and 30-s cycling powers. In addition, the thickness of the VL predicted the 5-s and 30-s cycling powers in a stepwise multiple regression analysis^1^. Ericson reported that that knee extensor contributed 39% of the cycling power^5,21^.

The present study found that various US characteristics of the RF, VL, and Gm were correlated with cycling power. These results remind about the importance of muscles in anterior thigh region to cycling, and are consistent with previous studies that employed surface EMG and needle EMG to reveal significant QF activation during pedaling. However, the correlation between muscle characteristics and cycling power differed between short- and long-distance cyclists in the present study. The muscle thicknesses of the VL measured at the 50% and 70% levels of the anterior thigh were strongly correlated with the cycling power in short-distance cyclists but not in long-distance cyclists. Since short-distance cyclists generate larger forces over short times and distances, the muscle architecture is likely to be strongly correlated with cycling power.

The fascicle length of the Gm measured at the 20% level on the posterior leg was strongly correlated with the 20-s cycling power in both the short- and long-distance cyclists. During cycling, the Gm maintains the propulsion provided by the VL and VM and delivers this propulsion to the pedal during the downstroke phase of pedaling (from 30° to 270°)^2,3,33^. However, the Gm does not produce a large amount of power itself during pedaling, although its activation increases with the pedaling rate, and a cyclist is required to generate higher forces at higher muscle contraction velocities (i.e., at a higher pedaling rates)^2,21^. Previous animal studies have revealed that the muscle fascicle length significantly influences the maximum shortening velocity^11,34^. Abe et al. found that the fascicles of the gastrocnemius are longer in sprinters than in distance runners. This would be advantageous for a faster sprinting and is also consistent with the fascicle length determining the maximum shortening velocity^17^. From these observations we can conclude that it is better for cyclists to aim for a shortening velocity through strong and rapid leg motions while pedaling at high speeds rather than exerting large forces, since cyclists have longer fascicles in their Gm.

Novel findings of this study are that the RF fascicle angle in long-distance cyclists and the fascicle length in short-distance cyclists were the main predictors of the 20-s cycling power in stepwise multiple regression analysis, accounting for 86% and 68% of the variance, respectively. It is known that muscle fascicle length plays a significant role in determining the maximum shortening velocity of the muscle^35^. Abe et al., reported that the fascicle length of selected locomotor muscles is significantly greater in elite sprinters than in elite distance runners^17^. Blazevich et al. conducted research applying specific resistance training to competitive athletes for 5 weeks and demonstrated that high-velocity training leads to an increase in fascicle length. The fascicle length at the proximal site greatly increased in the squat and sprint jump groups^36^. These results endorse the notion that different training interventions might produce inhomogeneous muscle hypertrophy along the length of a muscle.

It is assumed that structural heterogeneity allows muscles to represent different functional roles in their regions. For instance, while some regions are ideal for fast-speed muscle shortening or rapid force transfer on the tendon, other regions are ideal for high-power output. Hence, the different results from different training interventions can be deduced^36^. A previous study reported that track sprinters had longer fascicle lengths than long-distance runners, which highly correlated with their 100-m records^17^. As track sprint is an event that requires rapid contraction through short distances, short-distance cyclists would also need a longer fascicle length that could have a great impact on cycling power. As a result, the fascicle length seems to have been chosen as a significant variable to predict cycling power in short-distance cyclists who often perform high-speed training to improve their cycling performance.

When it comes to fascicle angle contribution for long-distance cyclists, Kordi et al. reported that peak power output strongly correlated with the fascicle angle of the vastus lateralis muscle (r = 0.81). The greater physiological cross-sectional area may have resulted from the greater fascicle angle of the vastus lateralis muscle, which leads to a higher force production capacity. However, the greater fascicle angle of the muscle could also result in loss of force transmission to the hard-contractile tissue (e.g., tendon) and/or decrease in fascicle length and, therefore, in shortening velocity^7^.

A long-distance cyclist usually contracts and relax muscles with high intensity and maintain them for a long duration. In addition, long-distance cyclists do not need as much rapid contraction as short-distance cyclists during training or competition, and fast force transmission is relatively rare. Therefore, it could be speculated that the fascicle angle could be a significant variable for predicting the cycling power for long-distance cyclists.

Whereas previous studies found greater muscle activation of the vastus group than the RF during cycling^37–39^, we found that the fascicle angle and length are strong predictors of the 20-s cycling power. This discrepancy might be attributable to differences in the included subjects and employed methodologies. Healthy, untrained, or sedentary subjects participated in the previous studies, and they used T2-weighted MRI and EMG to investigate muscles and their activation levels^37–39^. In contrast, highly trained cyclists participated in the present study, and we investigated the correlation between quantified aspects of muscle architecture and cycling power, without using EMG to also measure the muscle activation level. Since the muscle architecture can be changed (including optimized) by the type and duration of exercise, experimental studies can find different muscle contributions and activation levels. Therefore, further studies are required to determine the contributions of different muscles to cycling power and the performance in various subjects by combining the above-mentioned methods.

The RF is a biarticular muscle and functions as the primary hip flexor as well as acting as the primary knee extensors. This muscle is a fusiform muscle that generates one-third of the isometric torque about the hip joint^40^. Even though the RF is not a major force generator during pedaling, it is known to control the direction of the force produced by the monoarticular muscles (vastus group)^2,3,33^. Since the RF crosses the hip and knee joint, it functions differently in its proximal, middle, and distal parts. The proximal part is activated for hip flexion rather than knee extension compared to the other members of the vastus group. The muscles of the vastus group function during a relatively small phase (from 360° to 90°) during pedaling, while the RF functions over a wide range of pedaling phases. The proximal part of the RF was activated during the upstroke phase (between 200° and 90°), while its middle and distal parts were activated mainly during the upper-half phase (between 270° and 90°). The upstroke phase mainly contributes to hip flexion and is important for the transition of the force (i.e., propulsion phase) derived from the vastus group generated from the first half of the downstroke phase of pedaling^3^. The transition period is very brief, and so the RF needs to be able to contract rapidly so that the force induced during the downstroke phase of pedaling is not lost. Such a smooth and rapid pedaling technique would reduce the effort ‘wasted’ in bouncing, twitching, and weaving movements, thereby providing a more-solid base to deliver power from^41^.

Liao et al. reported that the hip joint provides most of the average power and a significant proportion of the power during the upstroke phase of pedaling (during hip flexion)^41^. For track sprint cyclists, the hip-joint power seems to dominate the pedaling action during both the maximum cadence and the submaximal cadence. Padulo et al. observed higher muscular activity for the RF during the start phase during testing on the track. The RF activity was higher in the standing posture than when seated, which is caused by both beginning earlier in the upward recovery phase and a delayed ending during the subsequent downstroke. These results suggest that the increasing RF activity is related to the higher power output in the standing posture during the sprint start phase^42^. Therefore, the fascicle length of the proximal area of the RF could contribute greatly to cycling performance in short-distance cyclists, and so coaches and trainers should be aware of the importance of the RF to predicting the cycling power as has been revealed by US.

The stepwise multiple regression analysis revealed that the fascicle angle of the RF was a major predictor of the 20-s cycling power for the long-distance cyclists. Since the muscle thickness did not differ significantly between long- and short-distance cyclists in this study, it can be expected that the muscle thickness or fascicle length contributes to the sports performance or cycling power in short-distance athletes, who need to be able to produce explosive forces over short periods. Franchi et al., reported that eccentric resistance training group showed increases in the fascicle length compared to the concentric training group. Based on the previous study, the eccentric strength could be helpful for short-distance cyclists who needs improvement in their fascicle length which leads greater force generating capacity^43,44^. On the other hand, the larger fascicle angle could contribute to sports performance in long-distance cyclists or distance runners, who need an endurance capacity with constant strength over long time periods^9^. Therefore, it can be proposed that a specific training or conditioning program should be established for long-distance cyclists that can improve endurance cycling ability, rather than only improving muscle thickness through high-resistance strength training.

The different contributions of the muscle architecture between short- and long-distance cyclists to cycling power could be supported by a previous study. Nagano A et al., when the location of the contractile tissue is closer to the joint, it is more suitable for the rapid development of force during the movements that act through a stiffer vehicle. Conversely, when the location of the contractile tissue is farther from the joint, it is more suitable for the overtime developing force during slower movements that encounter a more elastic vehicle^45^. For this reason, the proximal (30%) and middle (50%) RF were chosen as significant predictors of the cycling power for short- and long-distance cyclists, respectively, in the present study. This study used US to determine the muscle architecture in the anterior thigh region and the posterior leg region of cyclist, and we have obtained the key parameters that contribute to cycling power. Many previous studies have studied the muscle volume of the thigh region (including the QF, hamstring muscles, or trunk muscles as the iliopsoas muscle group) using MRI^7,12,13,20,29^, but few studies have used US to investigate the detailed architectural characteristics of cyclist muscles to clarify which variables predict the cycling power. US has advantages of faster scanning, real-time analysis, ease of analysis, and cheapness compared to MRI, which have resulted in US being used to predict muscle function and exercise performance since the 1990s in various sports fields. The present study found that the RF of the anterior thigh region was a strong predictor of the cycling power in both short- and long-distance cyclists. Even though several studies have provided information about muscle activity levels and the most-important muscles for improving pedaling technique and cycling performance, no previous US-based anatomical and physiological study has suggested which muscle architectural characteristics would be the most-valuable predictors of cycling power. The data obtained in this study made it possible to determine the correlation between the muscle architectural characteristics of the cyclists and cycling power. We believe that these data could also be useful in future studies that compare muscle characteristics and performance between different sports.

In conclusion, we compared the muscle architectural characteristics between short- and long-distance cyclists and clarified which muscle variables predict cycling power by measuring the muscle architectural characteristics using ultrasonography. The muscle architectural characteristics of the rectus femoris muscle showed differences between short- and long-distance cyclists. Furthermore, the rectus femoris fascicle angle in long-distance cyclists and the fascicle length in short-distance cyclists were the main predictors of 20-s cycling power. It can be proposed that a specific training or conditioning program should be established for long-distance cyclists to improve endurance cycling ability and fascicle angle of the rectus femoris, rather than to simply improve muscle thickness through high-resistance strength training. Eccentric training in addition to the stretching of the rectus femoris would enhance cycling performance for short-distance cyclist which is advantageous for faster sprinting and maximum shortening velocity.

## Methods

### Experimental approach to the problem

This study used a cross-sectional experimental design comprising two separate measurement sessions. The first measurement session was conducted to accumulate anthropometrics and US data. The cycling power measurement was completed following the US examination to lessen any edema related to muscle contraction that may have been induced during the cycling power measurements.

### Subjects

Twenty-four varsity cyclists (age, 20.7 ± 1.0 years; height, 174.5 ± 4.3 cm; weight, 72.2 ± 8.0 kg; mean ± SD) participated in this study, of whom 12 were short-distance cyclists and 12 were long-distance cyclists who had more than 5 years of cycling experience and had participated in cycling training for 24 ± 1 h per week. Each of the cyclists had either won first prize in a national college competition or had participated in an international college championship competition. Before the experiment, the subjects were informed of the purpose, methods, and risks of the study, and written informed consents were obtained from all of them. This study was approved by the Ethics Committee of Korea National Sport University, Seoul, South Korea (IRB No.: 20171026-016) and was performed in accordance with the Declaration of Helsinki.

### Ultrasonography scanning

**Figure 2:**
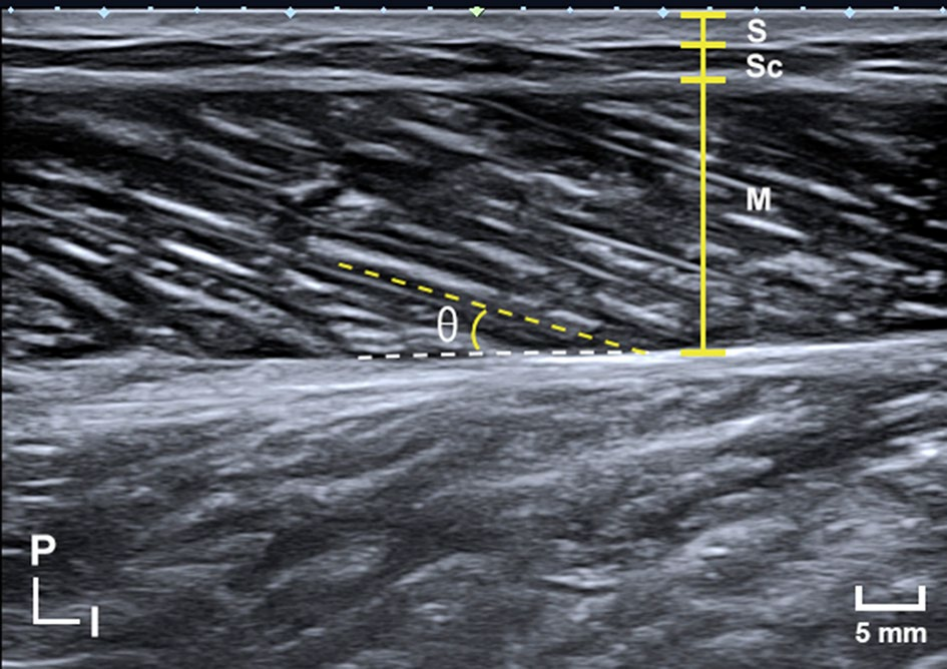
Representative US image shown the muscle thickness, fascicle angle, and fascicle length. The muscle thickness was measured between the superficial fascia and the deep fascia (white dashed line) or between the deep fascia and the bone, depending on the region. The fascicle angle (θ) was measured as the angle between the fascicle (yellow dashed line) and its superficial or deep fascia. When the fascicle was too long to measure from the origin to the insertion site, the fascicle length was estimated as muscle thickness/sin(θ). P, posterior; I, inferior; S, skin; Sc, subcutaneous tissue; M, muscle.

Real-time two-dimensional B-mode US (ECUBE 15, ALPINION Medical Systems, Seoul, Korea) with a 40-mm linear-array transducer (3.0–12 MHz; L3-12T, ALPINION Medical Systems) was used to measure muscle architecture parameters. The muscle thickness and fascicle angle were measured in the anterior thigh and posterior leg region (Fig. 2). The RF, VM, VL, and vastus intermedius (VI) in the anterior thigh region along with the Gm and Gl in the posterior leg region were also measured using US. The anatomical measurement sites are described in detail in Table 4.

**Table 4:** Description of the skeletal muscle characteristics measurement sites.

Muscle	Measurement site
RF VI	30%, 50%, and 70% level between the greater trochanter and lateral epicondyle of femur on the line connecting the anterior superior iliac spine and center of patella
VL	30%, 50%, and 70% level between the greater trochanter and lateral epicondyle of femur on the line parallel to the RF line passing through the lateral border of patella
VM	70% and 90% level between the greater trochanter and lateral epicondyle of femur on the line parallel to the RF line passing through the medial condyle of femur
Gm	20% and 30% level between fibular head and lateral malleolus on the perpendicular line passing through the medial border of the biceps femoris tendon at the popliteal fossa
Gl	20% and 30% level between fibular head and lateral malleolus on the perpendicular line passing through the lateral border of the semimembranosus tendon at the popliteal fossa

RF, rectus femoris; VI, vastus intermedius; VM, vastus medialis; VL, vastus lateralis; Gm, medial head of gastrocnemius; Gl, lateral head of gastrocnemius.

**Figure 3:**
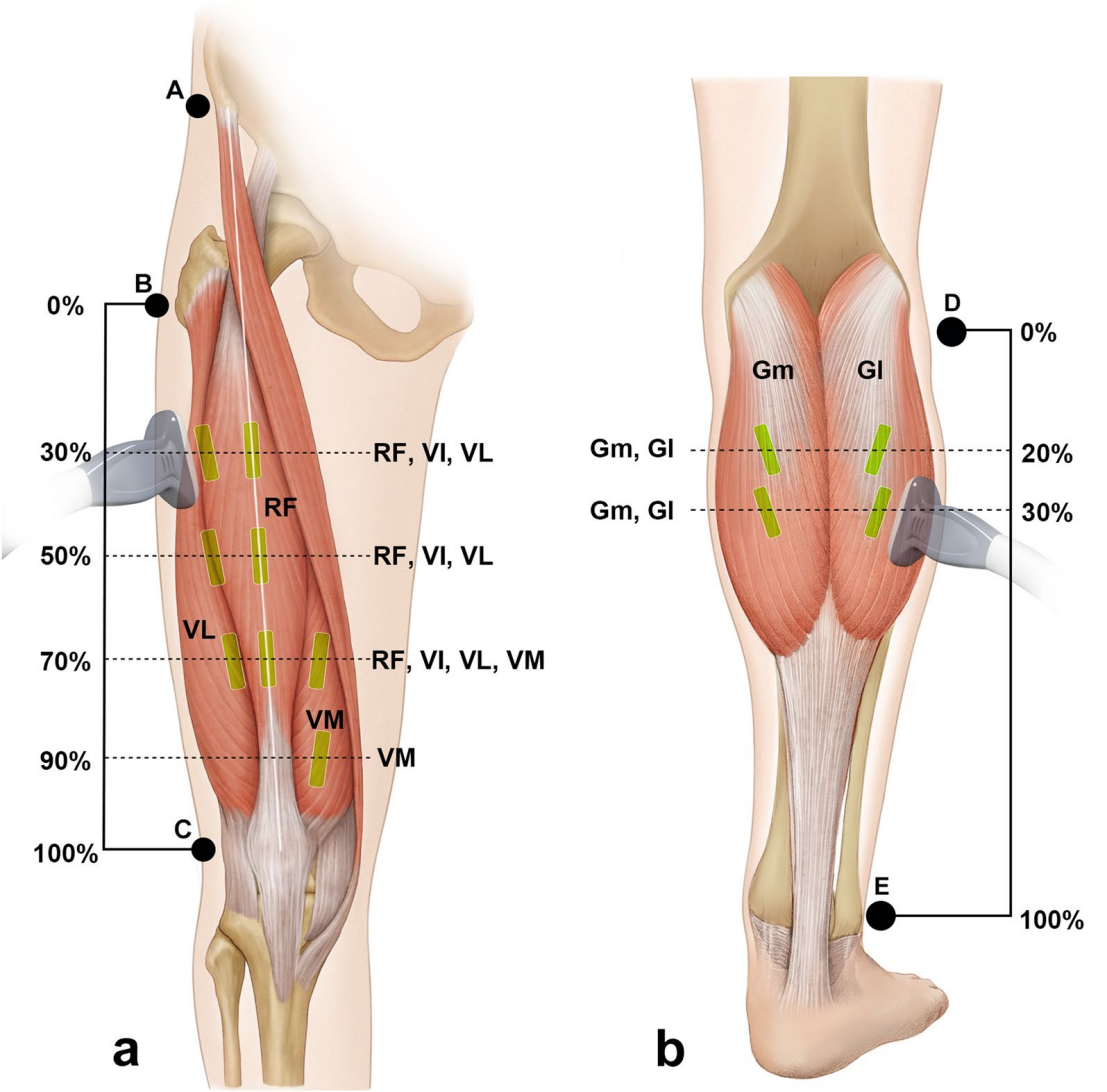
Illustration depicting the measurement sites for the skeletal muscle architectural characteristics. US images were acquired at lengths equivalent to the 30%, 50%, 70%, and 90% levels of the thigh length measured from the greater trochanter to the lateral epicondyle of the femur (**a**), and to the 20% and 30% levels of the leg length measured from the fibular head to the lateral malleolus (**b**). The white solid line in A indicates the vertical reference line connecting the anterior superior iliac spine and the center of the patella. The transducer was positioned at the intersection points between the vertical reference line and each measured level (i.e., 30%, 50%, 70%, and 90%). Based on this line, the muscle architectural characteristics of the VL were quantified parallel to the RF and to the VI lines passing through the lateral border of the patella. A, anterior superior iliac spine; B, greater trochanter; C, lateral epicondyle of the femur; D, fibular head; E, lateral malleolus; RF, rectus femoris; VL, vastus lateralis; VM, vastus medialis; VI, vastus intermedius; Gl, lateral head of the gastrocnemius; Gm, medial head of the gastrocnemius.

The muscle thickness was defined as the distance between the superficial fascia and the deep fascia or between the deep fascia and the bone, depending on the region. The fascicle angle was defined as the angle between the fascicle and deep fascia or the bone, depending on the region. Muscles show different morphologies in the anterior thigh, including the hypertrophic region and the fascicle length of the muscle belly. Therefore, the muscle architectures of the RF, VI, and VL (including the muscle thickness and fascicle angle) were measured at the 30%, 50%, and 70% levels between the greater trochanter and the lateral epicondyle of the femur at the anterior thigh (Fig. 3). The fascicle angle of the VM was not measured because its fascicle was highly curved. The muscle thickness and fascicle angle of the Gm and Gl were measured at proximally 20% and 30% between the fibular head and the lateral malleolus.

The fascicle from the origin to the insertion site was too long to measure using US (Fig. 2), and so its length was calculated as muscle thickness/sin (fascicle angle), where the muscle thickness and fascicle angle were measured using image analysis software (Image J, National Institutes of Health, Bethesda, MD, USA).

The US penetration depth was adjusted to 4–8 cm depending on the individual subject. The measurements were made with the subject in a supine position on the examination table with the knee joint at an angle of 90°. The US probe was placed perpendicular to the skin, and a water-soluble gel was applied to the skin to obtain a high-resolution image without losing the detailed anatomical features of the muscles. Each measurement site was clearly marked on the skin surface with a surgical pen to ensure that the position where the probe was placed was consistent across repeated scans. The use of the gel meant that the US probe could be positioned just above the skin surface at each landmark without pressure being applied to the skin.

### Cycling power

A cycle ergometer (Wattbike, Nottingham, UK) that is often used by cyclists was used to measure the cycling power. Each subject adjusted the heights of the handlebar and saddle and the distance from the saddle to the handle to suit their individual body dimensions. Before measuring the cycling power, the cyclists were instructed to warm up for a few minutes using their own bikes on an unloaded cycle roller. The test variable for measuring cycling power was the mean anaerobic power over a period of 20 s. The subjects were asked to perform at their maximum effort level during these tests.

### Statistical analyses

All statistical analyses were conducted using standard software (SPSS version 23.0 for Windows, SPSS, Chicago, IL, USA). The probability cutoff for statistical significance was *p* < 0.05. Two examiners measured the muscle thickness and fascicle angle twice using US, and the differences between the values obtained by the two examiners were used to determine the intraclass and interclass correlation coefficients. Correlation coefficients of < 0.4, 0.4–0.6, 0.6–0.75, and 0.75–1.00 are considered to indicate poor, moderate, good, and excellent reliability, respectively, and in the present study statistical analyses were conducted when coefficients of 0.75–1.00 were achieved.

The differences in muscle thickness, fascicle angle, and fascicle length between measured sites for each muscle in short- and long-distance cyclists were determined using One-way analysis of variance (ANOVA). Tukey’s post-hoc test was performed when a significant difference was detected in any ANOVA, in which the data conformed to a normal distribution (RF and VL, where the scanning was performed at three sites). Paired t-tests with Bonferroni adjustment were used for the data of VM, Gm, and Gl where the scanning was performed at two sites. The differences in muscle thickness, fascicle angle, and fascicle length between short- and long-distance cyclist were determined using an independent t-test. However, the Kruskal–Wallis test and a Mann–Whitney test with Bonferroni correction (*P* value of 0.017 was considered to indicate statistical significance) were used to determine differences among the three scanning sites for the data that did not conform to a normal distribution (i.e., VI).

Pearson’s correlations were used to evaluate the relationships between the muscle architectural characteristics (muscle thickness, fascicle angle, and fascicle length) and the 20-s mean anaerobic power. Stepwise multiple regression analyses were performed to establish a predictive model of cycling power (mean anaerobic power) for each group (short- and long-distance cyclists). The muscle thickness, fascicle angle, and fascicle length at all measured sites for each muscle were used as the independent variables.

## Abbreviations

EMG: Electromyography
US: Ultrasonography
QF: Quadriceps femoris
MRI: Magnetic resonance imaging
RF: Rectus femoris
VM: Vastus medialis
VL: Vastus lateralis
VI: Vastus intermedius
Gm: Medial head of the gastrocnemius
Gl: Lateral head of the gastrocnemius
ANOVA: One-way analysis of variance
